# Topological congruence between phylogenies of *Anacanthorus* spp. (Monogenea: Dactylogyridae) and their Characiformes (Actinopterygii) hosts: A case of host-parasite cospeciation

**DOI:** 10.1371/journal.pone.0193408

**Published:** 2018-03-14

**Authors:** Rodrigo J. da Graça, Thomaz M. C. Fabrin, Luciano S. Gasques, Sônia M. A. P. Prioli, Juan A. Balbuena, Alberto J. Prioli, Ricardo M. Takemoto

**Affiliations:** 1 Departamento de Ciências Biológicas, Programa de Pós-Graduação em Ecologia de Ambientes Aquáticos Continentais, Universidade Estadual de Maringá, Núcleo de Pesquisas em Limnologia, Ictiologia e Aquicultura, Maringá, Paraná, Brazil; 2 Programa de Pós-Graduação em Biologia Comparada, Universidade Estadual de Maringá, Maringá, Paraná, Brazil; 3 Instituto de Ciências Biológicas, Médicas e da Saúde, Universidade Paranaense, Umuarama, Paraná, Brazil; 4 Cavanilles Institute of Biodiversity and Evolutionary Biology, Science Park, University of Valencia, Paterna, Valencia, Spain; University of Pretoria, SOUTH AFRICA

## Abstract

Cophylogenetic studies aim at testing specific hypotheses to understand the nature of coevolving associations between sets of organisms, such as host and parasites. Monogeneans and their hosts provide and interesting platform for these studies due to their high host specificity. In this context, the objective of the present study was to establish whether the relationship between *Anacanthorus* spp. with their hosts from the upper Paraná River and its tributaries can be explained by means of cospeciation processes. Nine fish species and 14 monogenean species, most of them host specific, were studied. Partial DNA sequences of the genes *RAG1*, *16S* and *COI* of the fish hosts and of the genes *ITS2*, *COI* and *5*.*8S* of the parasite species were used for phylogenetic reconstruction. Maximum likelihood phylogenetic trees of the host and parasite species were built and used for analyses of topological congruence with PACo and ParaFit. The program Jane was used to estimate the nature of cospeciation events. The comparison of the two phylogenies revealed high topological congruence between them. Both PACo and ParaFit supported the hypothesis of global cospeciation. Results from Jane pointed to duplications as the most frequent coevolutionary event, followed by cospeciation, whereas duplications followed by host-switching were the least common event in *Anacanthorus* spp. studied. Host-sharing (spreading) was also identified but only between congeneric host species.

## Introduction

Cophylogenetic studies have been pursued by researchers since the 19th century. Many works have focused on testing specific hypotheses to determine which coevolutionary events gave rise to the patterns of association between hosts and parasites observed [1–6].

In the aquatic realm, monogeneans and their hosts have been widely used in coevolutionary studies, mostly due to their usually high host specificity [7–9]. In fact, some monogeneans are so specific that have been proposed as a tool to identify their host species [10]. This tight host specificity can be interpreted as evidence of cospeciation [11–13], but note that host specificity does not always result from cospeciation [14].

In parasites, four types of evolutionary events, which can act concurrently in a given parasitic taxa, may lead to host specificity: coespeciation, in which the parasite speciates following or along with host speciation; duplication, in which the parasite speciates within the same host species; failure to diverge (also known as lineage sorting) in which the parasite fails to diverge and is lost after host speciation [1–2]; and host switching, where the parasite is able to colonize and speciate in a new host unrelated to the original one [14–16]. In this case, the parasite undergoes speciation as adaptation to the physiological and morphological traits of the new host, thereby providing a new resource to be exploited. However, colonization of a new host does not necessarily lead to speciation resulting in host-sharing (also known as spreading) [17–19], a process that leads to generalist parasites.

Contrary to the view that specificity in monogeneans is entirely accounted for by host-parasite evolutionary relationships, it has been pointed out that ecological factors [20], together with high speciation rates and host-switching opportunities [1, 15] can act concurrently.

In fact, different coevolutionary studies of monogeneans and their fish hosts, such as *Lamellodiscus* on Sparidae (4), *Thaparocleidus* on Pangasiidae [21], *Cichlidogyrus* on Cichlidae [22–24], *Dactylogyrus* on Cyprinidae [25, 26], and *Gyrodactylus* on Gobiidae [27] and Salmonidae [28], have shown that host switching and duplication are the most important evolutionary events in parasite diversification and that cospeciation is relatively rare in these host-parasite systems [4, 22].

*Anacanthorus* Mizelle and Price, 1965 is one of the more speciose genera of gill monogeneans of freshwater Characiformes in the Neotropical region [29, 30]. Up to 2013, some 70 species had been recorded only in South America [30]. However, recent surveys, check-lists [31, 32] and species descriptions [33, 34] indicate that the actual number of species in the region is probably much higher.

The distribution and colonization of many species of *Anacanthorus* on their hosts might have been influenced by the evolutionary history of the Characiformes in the Neotropical region [35]. This makes this host-parasite system very attractive for biogeographical and coevolutionary studies, as revealed by work on *Anacanthorus* spp. on serrasalmids in Northern Brazil [36].

Therefore, the present effort aims at establishing the coevolutionary processes linking species of *Anacanthorus* with their hosts in the upper Paraná River and its tributaries. Specifically we intend to assess the role of cospeciation in the diversification of this genus as opposed to duplication and host-switching events that seem to predominate in many fish-monogenean systems.

## Materials and methods

### Study area, host and parasite collection

**Fig 1:**
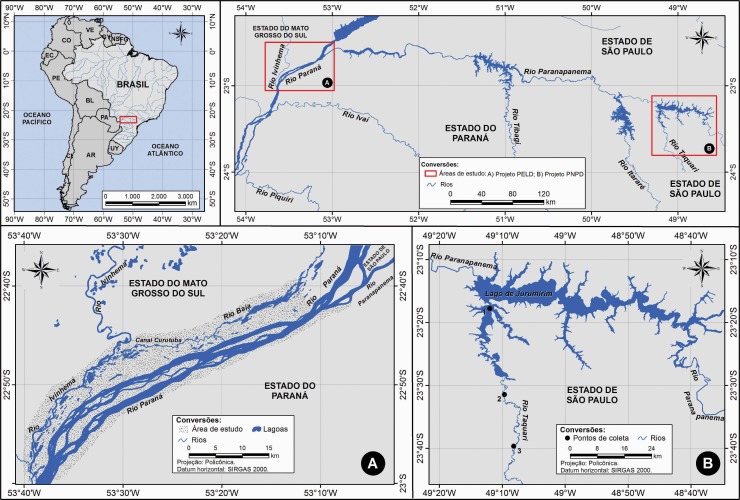
Sampling sites of fish in the Paraná River and its tributaries between 2012 and 2015. A) Project PELD area with rivers Ivinheima (22°47’S—53°32’W), Baia (22°43’S—53°17’W) and Paraná (22°45’S—53°15’W) in the flood plain of the upper Paraná River. B) Sampling sites of *Salminus hilarii* in the Taquari River: Site 1 (23°17’48. 23” S; 49°11’56.74” W); Site 2 (23°39’42.12” S; 49°08'08.42" W); Site 3 (23°31'28.82" S; 49°09'37.04" W).

The study area belongs to the flood plain of the upper Paraná River, an environmentally preserved area extending across the states of Paraná (PR) and Mato Grosso do Sul (MS), Brazil. The sampling sites correspond to those used in project LTEP–CNPq (Long-Term Ecological Projects)–Site 6 (Fig 1A).

Additional specimens of *Salminus hilarii* Valenciennes, 1850 were obtained in the Taquari River, because those collected in the Paraná River were devoid of parasites. These fish were captured by researchers of the Laboratório de Parasitologia de Animais Silvestres (LAPAS), Department of Parasitology, Instituto de Biociências da Universidade Estadual Paulista (UNESP), Botucatu, State of São Paulo. This river, located in the State of São Paulo, is a left-tributary of the Paranapanema River and belongs to the Paraná River basin (Fig 1B).

Fish were collected between 2012 and 2015 with a permit from the Instituto Chico Mendes–ICMbio (SISBIO 22442–1). Fish were collected with gillnets (2.4 to 16 cm mesh) and fishing rods and transported to the laboratory. They were then anaesthetised and sacrificed with benzocaine 10%, according to the regulations of animal welfare approved by the Ethics Commission of the Universidade Estadual de Maringá, (CEUA123/2010). The fish species were identified according to Graça and Pavanelli [37].

Nine fish species of Characiformes were collected: *Metynnis lippincottianus* (Cope, 1870) “pacu cd”, *Piaractus mesopotamicus* (Holmberg, 1887) “pacu”, *Serrasalmus maculatus* Kner, 1858 “piranha”, *Serrasalmus marginatus* Valenciennes, 1837 “piranha”, *Hoplias malabaricus* (Bloch, 1794) “traíra”, *Erythrinus erythrinus* (Bloch and Schneider, 1801) *“*jeju mole*”*, *Hoplerythrinus unitaeniatus* (Spix and Agassiz, 1829) *“*jeju*”*, *Salminus hilarii* “tabarana” and *Salminus brasiliensis* (Cuvier, 1816) “dourado”.

The species were chosen according to previous records of *Anacanthorus* spp. in the study area [38], or data from host-parasite checklists [30]. The range of host species included members of different genera and families of Characiformes in order to test whether the species of *Anacanthorus* occurring on phylogenetically related hosts are also related.

Fish specimens were thawed and their gills removed and kept refrigerated for preservation of parasites. Each gill arch was examined individually for parasites in a Petri dish with chilled water under a stereo microscope. The specimens of *Anacanthorus* collected were transferred to a drop of tap water on a microscope slide and examined under a cover slip with an Olympus CX31 microscope for species identification.

All parasite specimens were photographed with a Sony Cyber-Shot DSC W5 camera fitted to the microscope. Pictures of the male copulatory complex were used for species identification given that this is the main diagnosis character in *Anacanthorus* [29]. These pictures were named and archived for further reference. Then the specimens were removed from the slide and transferred to a 2 mL microtube with 20 μL of ultrapure water for subsequent DNA extraction.

The species of *Anacanthorus* were identified according to Boeger et al. [39], Cohen et al. [30, 40] and Leão et al. [33]. The forms that could not be identified to species level possibly represent new species to science that will be described in due course.

### DNA extraction, PCR and sequencing

The parasite specimens were placed individually in microtubes and DNA extraction was performed with a commercial kit, ReliaPrep^TM^ gDNA Tissue Miniprep System, Promega. DNA from fish specimens of *Metynnis lippincottianus*, *Erythrinus erythrinus* and *Serrasalmus marginatus* was also extracted because no sequences of interest were previously available in GenBank. Fish DNA was extracted with a Wizard Genomic DNA Purification kit from Promega. Both extractions were performed according to the manufacturer’s protocol. Extracted DNA was kept in labeled microtubes at– 20°C.

Polymerase chain reaction (PCR) was run in a Applied Biosystems® ProFlex™ PCR System thermocycler in solution containing buffer Tris-KCl [20 mM Tris-HCl (pH 8.4), 50 mM KCl], MgCl2 (1.87 mM), primers (2.5 pmoles), dNTPs (0.5 mM), Taq DNA Polymerase Platinum–Invitrogen® (1 U), extracted DNA (4–6 μL) and water q.s. 20 μL.

The Cytochrome C oxidase I (*COI*) mitochondrial gene of parasites was amplified with primers Trem Co1F (5´-TTTCGTTGGATCATAAGCG-3´) and Trem Co1R (5´- GCAGCACTAAATTTACGATCAAA-3´) developed by Bonett et al. [41]. The amplification reaction consisted of 35 cycles of denaturation at 94°C for 30 seconds, annealing at 44°C for 30 seconds, extension for 2 minutes at 72°C and final extension for 7 minutes at 72°C. *COI* genes of *M*. *lippincottianus* and *E*. *erythrinus* were amplified with primers L6448 (5´-TCGACTAATCATAAAGATATCGGCAC-3´) and H7152 (5´-CACCTCAGGGTGTCCGAARAAYCARAA-3´) designed by Ivanova et al. [42]. Amplification was performed in 35 cycles, with denaturation at 95°C for 1 minute, annealing at 52°C for 40 seconds, extension for 1 minute at 72°C and final extension for 10 minutes at 72°C.

Internal transcribed spacers (*ITS1* and *ITS2*) rDNA and *5*.*8S* rDNA of the parasites were amplified with primers Bd1 (5′-GTCGTAACAAGGTTTCCGTA-3′) and Bd2 (5′-TATGCTTAAATTCAGCGGGT-3′) devised by Luton et al. [43]. The thermocycling profile consisted of 30 cycles, with denaturation at 94°C for 30 seconds, annealing at 56°C for 30 seconds, extension for 1 minute at 72°C and final extension for 5 minutes at 72°C. The nuclear *RAG1* gene of *S*. *marginatus* was amplified with primers RAG1-4063R (5´- TTCTGNARRTACTTGGARGTGTAWAGCCA-3´) and RAG1-3098F (5´- TGTGCCTGATGYTYGTDGAYGART-3´) designed by Li and Ortí [44]. The amplification conditions were in 41 cycles, with denaturation at 94°C for 4 minutes in the first cycle and 15 seconds in the rest, annealing at 55°C for 30 seconds, extension for 2 minutes at 72°C and final extension final for 5 minutes at 72°C.

The PCR amplicons were purified in Polyethylene glycol 8000 – 2M 80% NaCL, using 40 μL of PEG-NaCL and about 17 μL of the amplified DNA sample as per Rosenthal et al. [45].

For sequencing, individual reactions were run with the same primers used in the corresponding PCRs. The samples were prepared in a final volume of 6 μL, following the manufacturer’s instructions of the BigDye Terminator kit. Sequencing was carried out with a ABI3730 automatic sequencer at the Central Laboratory of High Performance Technologies, Universidade de Campinas, Brazil. The sequences obtained were edited with BioEdit 7.2.5 [46].

Additional sequences of *RAG1*, *16S* and *COI* for further phylogenetic analysis were retrieved from GenBank (Table 1). All sequences were aligned with MUSCLE [47] in MEGA 6 [48].

**Table 1:** GenBank accession numbers of the DNA sequences of genes RAG1, COI and 16S of fish hosts and associated species of *Anacanthorus* detected in the present effort on each fish species.

Hosts	RAG1	COI	16S	Parasite species
*S*. *brasiliensis*	HQ289336	KU288818	HQ171437	*Anacanthorus bicuspidatus* *Anacanthorus contortus* *Anacanthorus douradensis* *Anacanthorus parakruidenieri*
*S*. *hilarii*	KF780113	JN989211	KF780006	*Anacanthorus bicuspidatus* *Anacanthorus contortus*
*P*. *mesopotamicus*	HQ289217	HQ420833	HQ171315	*Anacanthorus penilabiatus* *Anacanthorus toledoensis*
*M*. *lippincottianus*	HQ289265	MF063324*	HQ171364	*Anacanthorus* sp. 6 *Anacanthorus* sp. 7
*S*. *marginatus*	MF063323*	JN989235	DQ384743	*Anacanthorus* sp. 1 *Anacanthorus* sp. 2
*S*. *maculatus*	HQ289189	HQ289242	HQ171285	*Anacanthorus* sp. 1 *Anacanthorus* sp. 2
*H*. *unitaeniatus*	HQ289309	HQ289242	HQ171408	*Anacanthorus* sp. 4 *Anacanthorus* sp. 5
*H*. *malabaricus*	HQ289248	JN988909	JX470044	*Anacanthorus* sp. 3
*E*. *erythrinus*	HQ289242	MF063325*	HQ171340	*Anacanthorus* sp. 8

* Sequences obtained in the present study

### Phylogenetic and cophylogenetic analyses

The best nucleotide substitution model was selected with jModelTest [49]. Phylogenetic analyses were carried out with concatenated sequences of genes *RAG1*, *COI* and *16S* for the hosts and *COI* and partial *ITS* region, (*5*.*8S* + *ITS2*) for the parasites. The Maximum Likelihood method was used for phylogenetic reconstruction of *Anacanthorus* spp. and their hosts. In both cases, the GTR + Γ substitution model was chosen. The analyses were implemented with RAxML [50] using the rapid bootstrap algorithm with 1,000 resamples. The reconstructions followed the partitions recommended by PartitionFinder [51], also considering the subpartitions of codons of the genes *COI* and *RAG1* for the hosts, and *COI* for the parasites. The partitions used were *COI* (1^st^, 2^nd^) + *RAG1* (1^st^, 2^nd^); *RAG1* (3^rd^); *COI* (3^rd^); *16S* and *COI* (1^st^); *COI* (2^nd^), *COI* (3^rd^); *ITS2*; *5*.*8S*, for hosts and parasites, respectively.

GenBank sequences of *Carassius auratus* Linnaeus, 1758 (Cyprinidae) (KJ474758.1, HQ654690.1 and LC097877.1) and of *Gyrodactylus gurleyi* Price, 1937 (Gyrodactylidae) (KU659806.1 and AJ001842.1) were used to represent the outgroups in the reconstruction of the fish and parasite phylogenies, respectively. All sequences of the parasite species obtained for the *COI*, *ITS2* and *5*.*8S* markers have been deposited in GenBank (MF034464—MF034491).

In order to test the congruence between the host and parasite phylogenies, ParaFit [52] and *Procrustean Approach to Cophylogeny* (PACo) [53, 54] were used. Both analyses were performed in R [55].

The patristic distance matrices of the host and parasite trees and a binary matrix describing the associations between each host and parasite species (0, no association; 1, association) were used as input in these analyses [52,53]. The congruence between the host and the parasites phylogenies was tested by means of 10,000 random permutations of the binary matrix following the randomization schemes described in Legendre et al. [52] and Balbuena et al. [53] for ParaFit and PACo, respectively. The contribution of each individual host-parasite link to the total phylogenetic congruence was tested with ParaFitLink1 and ParaFitLink2 [52] and assessed by establishing the contribution of the squared residual associated with each host-parasite link to the total sum of squared residuals in PACo [53,54]. In all tests, the significance level considered was 0.01.

Jane v4 [19] was used to determine which coevolutionary events likely accounted for the patterns of host-parasite associations observed. This analysis assigns a range of costs to each coevolutionary event and attempts to identify which scenario minimizes the costs. Jane considers the following events: duplication, loss, failure to diverge, duplication followed by host switching, (see Introduction for definition of terms). The analysis was run with the costs recommended by the program [19] for 300 generations to obtain the best solution.

## Results

### Phylogenetic analysis

Continuous alignment of all genes was achieved following manual editing. The aligned sequence lengths were 454pb and 500pb for *COI* of hosts and parasites, respectively, 525pb for 16S, 1016pb for *RAG1* and 460pb for *ITS* (156pb of 5.8S + 304pb partial *ITS2*).

In general, bootstrap nodal support was strong in both the host and parasite phylogenies. Exceptions were nodes within the fish families Erythrinidae and Serrasalmidae (37%), nodes relating *Anacanthorus* spp. parasitizing serrasalmids (41%) and briconids and eritrinids (42%).

**Fig 2:**
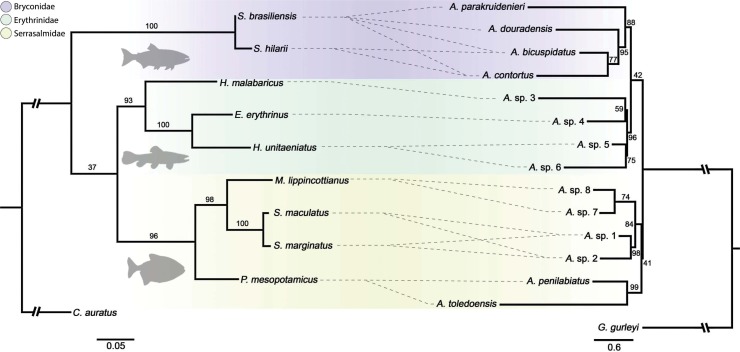
**Tanglegram depicting the relationship between nine species of Characiformes (left) and 14 species of *Anacanthorus* (right)**. Background colours correspond to the three families of hosts represented and their respective parasite species. Dotted lines indicate the host-parasite associations. Abbreviations of *Anacanthorus* spp.: A.sp. 1 = *Anacanthorus* sp. 1; A.sp. 2 = *Anacanthorus* sp. 2; A.sp. 3 = *Anacanthorus* sp. 3; A.sp. 4 = *Anacanthorus* sp. 4; A.sp. 5 = *Anacanthorus* sp. 5; A.sp. 6 = *Anacanthorus* sp. 6; A.sp. 7 = *Anacanthorus* sp. 7; A.sp. 8 = *Anacanthorus* sp. 8.

The tanglegram indicated high congruence between the host and parasite phylogenies (Fig 2). The *Anacanthorus* species were grouped in three large clades, each associated with a host family. Closely related hosts were parasitized by sister clades of *Anacanthorus*. For instance, *Anacanthorus penilabiatus* and *A*. *toledoensis*, occurring both on *Piaractus mesopotamicus*, formed a sister clade with *Anacanthorus* sp.1, *Anacanthorus* sp.2, *Anacanthorus* sp.7 and *Anacanthorus* sp.8, associated with three additional serrasalmids, *Metynnis lippincottianus*, *Serrasalmus marginatus* and *S*. *maculatus* (Fig 2).

### Congruence analyses

Both PACo and ParaFit provided significant evidence for phylogenetic congruence between the species of *Anacanthorus* studied and their hosts (PACo m^2^_XY_ = 0.621, *p* = 0.0000; ParaFitGlobal = 11.68181, *p* = 0.0001), rejecting the null hypotheses of the similarities between the phylogenies having arisen just by chance.

**Fig 3:**
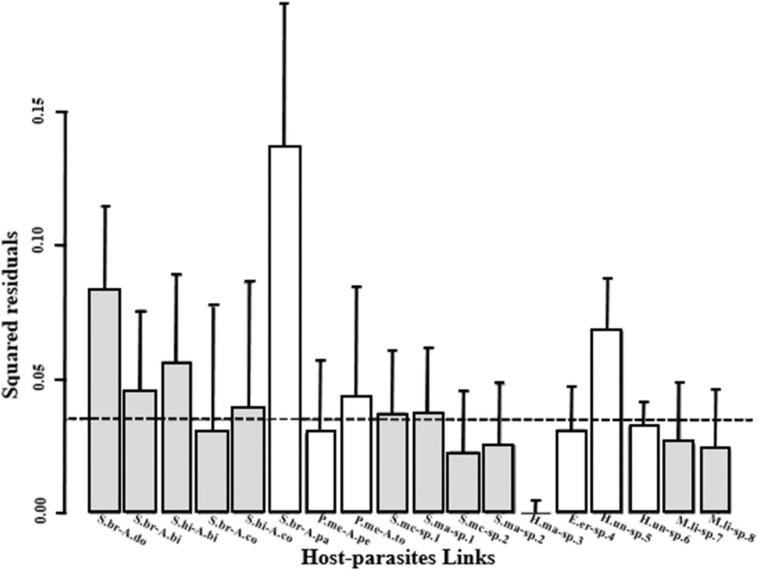
Contribution of individual associations to global phylogenetic congruence of *Anacanthorus* spp. and their hosts. The columns represent the squared residual of each association contributing to the total sum of squared residuals computed by PACo for patristic distances derived from the phylogenies shown in Fig 2). Error bars correspond to +95% confidence intervals. The median squared residual value is indicated by the stippled line. Host-parasite associations whose 95% squared residual confidence interval falls above the median value probably represent non-congruent associations. Grey bars represented significant coevolving links according to ParaFitLink 1 and ParaFitLink 2 at *α* = 0.01. Abbreviations of Characiformes names: Bryconidae (S.hi = *Salminus hilarii*; S.br = *Salminus brasiliensis*); Serrasalmidae (P.me = *Piaractus mesopotamicus*; S.mc = *Serrasalmus maculatus*; S.ma = *Serrasalmus marginatus*; M.li = *Metynnis lippincottianus*); Erythrinidae (H.ma = *Hoplias malabaricus*; E.er = *Erythrinus erythrinus*; H.un = *Hoplerythrinus unitaeniatus*). Abbreviations of parasite names: A.bi *= A*. *bicuspidatus*; A.co = *A*. *contortus*; A.do = *Anacanthorus douradensis*; A.pa = *Anacanthorus parakruidenieri*; A.sp. 1 = *Anacanthorus* sp. 1; A.sp. 2 = *Anacanthorus* sp. 2; A.pe = *Anacanthorus penilabiatus*; A.to = *Anacanthorus toledoensis*; A.sp. 3 = *Anacanthorus* sp. 3; A.sp. 4 = *Anacanthorus* sp. 4; A.sp. 5 = *Anacanthorus* sp. 5; A.sp. 6 = *Anacanthorus* sp. 6; A.sp. 7 = *Anacanthorus* sp. 7; A.sp. 8 = *Anacanthorus* sp. 8.

There was no agreement between PACo and ParaFit in the analysis of individual host-parasite associations. In PACo, inspection of the squared residuals of each host-parasite association indicated that the confidence intervals of those corresponding to *S*. *brasiliensis–A*. *parakruedenieri*, *S*. *brasiliensis–A*. *douradensis* and *H*. *unitaeniatus–Anacanthorus* sp. 5 did not include the median squared residual value (Fig 3). In contrast, the analysis with ParaFitLink 1 and ParaFitLink 2 pointed to significant support for the associations within Bryconidae and Serrasalmidae, suggesting cospeciation events with their associated species of *Anacanthorus* (Fig 3).

**Fig 4:**
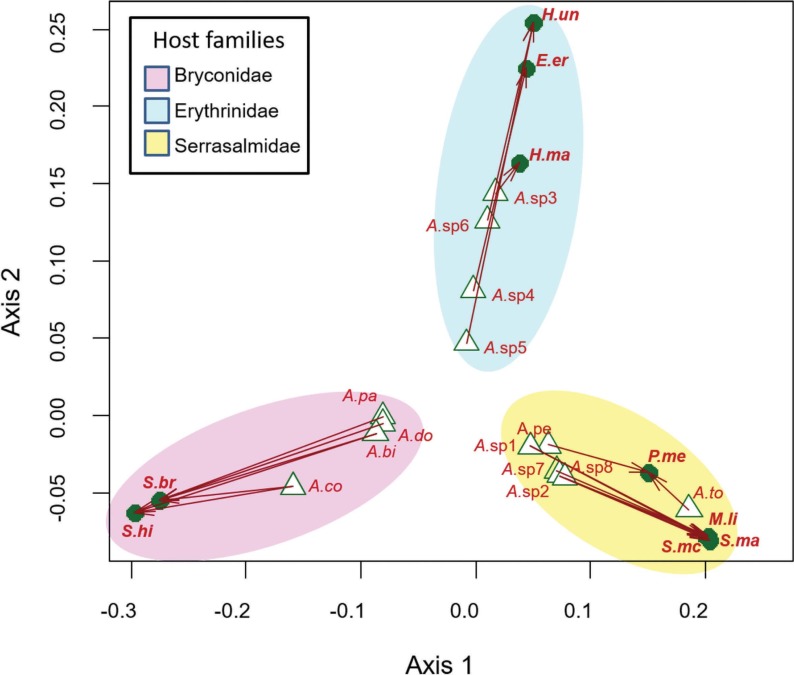
Procrustean superimposition plot which minimizes the differences between the principal coordinates of patristic distances of *Anacanthorus* spp. and their Characiformes hosts. For each vector, the starting point (triangles) represent the configuration of *Anacanthorus* spp. and the arrowhead (points) the configuration of the corresponding hosts. The vector length represents the global fit (residual sum of squares) which is inversely proportional to the topological congruence. Host associations were grouped according to host families. Abbreviations of species names are the same as in Fig 3.

The procrustean superimposition plot suggested three groups of host–parasite associations (Fig 4). One group is composed of *Anacanthorus* species associated with Bryconidae fish. The second group is composed of *Anacanthorus* species associated with Erythrinidae fish. And a third group of *Anacanthorus* species parasites of Serrasalmidae.

**Fig 5:**
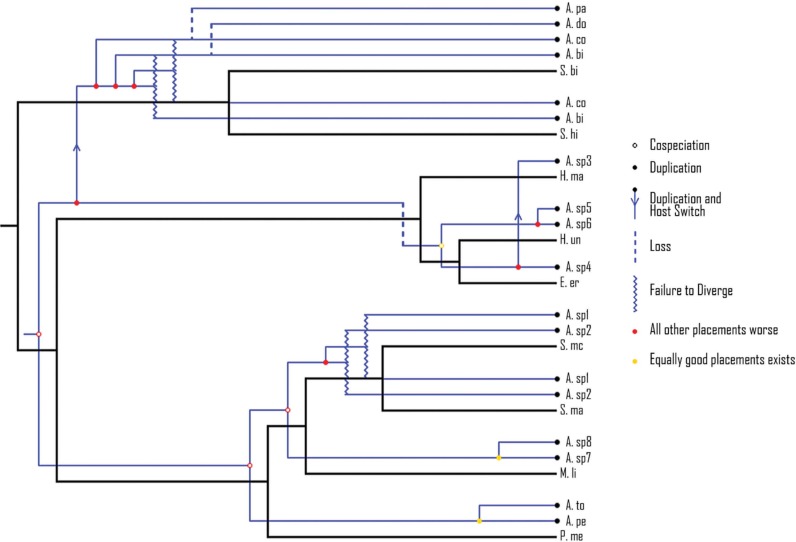
One of the 60 possible cophylogenetic reconstructions of *Anacanthorus* spp. and their hosts returned by Jane. Black branches (independent) represent the host phylogeny. Blue branches (dependent) correspond to the parasite phylogeny. The events and costs considered were cospeciation (0), duplication (1), duplication followed by host switching (2), loss (1) and failure to diverge (1). Abbreviations of species names are the same as in Fig 3.

The smallest cost scheme returned by Jane was 18, corresponding to four cospeciation, seven duplications, two duplications followed by host switch, three losses and four failures to diverge (Fig 5).

## Discussion

This is the first study using molecular markers to study coevolutionary processes in *Anacanthorus* spp. and their hosts in South America. Both PACo and ParaFit supported the hypothesis of phylogenetic congruence between *Anacanthorus* spp. and their hosts, which indicates a common coevolutionary history in these organisms.

However, the analysis of individual host-parasite associations by PACo and ParaFit rendered conflicting results and it can be concluded that there is no clear node-to-node correspondence between the phylogenies tested. In fact, Jane indicated that duplications were probably the most widespread evolutionary event in the diversification of *Anacanthorus*, followed by cospeciations.

Jane rendered a coevolutionary scenario (Fig 5) where cospeciation events occurred mostly at the upper level within fish families and genera, which might well correspond to biogeographic events, this is consistent with what is widely known in host-parasite coevolution and in patterns of host specificity in freshwater fishes [24, 27]. So, vicariance, or even dispersion, leading to speciation in the hosts might have driven speciation in their parasites. These recent cospeciation events could also have favoured host-sharing due to the phylogenetical proximity of the hosts involved. Thus, *Salminus brasiliensis* and *S*. *hilarii* share two of five species occurring on these hosts, and *Serrasalmus maculatus* and *S*. *marginatus* are parasitized by the same species of *Anacanthorus*.

In Monogenea it has been postulated that cospeciation should be expected at high host taxonomic levels, such as families and genera [15]. Thus, the phylogenetic associations between monogeneans and their hosts would be driven by historical events, such as immunological or morphological barriers, acting at these taxonomic levels [4]. Studies of interaction networks between gill monogeneans and fish in the Neotropical region evidenced a restricted composition of the monogenean fauna influenced by the phylogenetic relationship of their hosts and their geographic distribution [35]. Our results conform with this scenario since each fish species harboured a unique composition of *Anacanthorus* spp. and phylogenetically close hosts shared some of the parasite species.

In addition, the diversification patterns in *Anacanthorus* in relation to their hosts have some similarities with those reported in other monogeneans. For example, in species of *Cichlidogyrus* on ciclhids, it has been suggested that their diversification could be accounted for by isolation due to host specialization, followed by duplications resulting in a diversity of parasites larger than that of their hosts [24]. This seems a plausible scenario for the species of *Anacanthorus* studied, given that, of the nine host species, only *Hoplias malabaricus* and *Erythrinus erythrinus* were parasitized by a single species and the number of species of *Anacanthorus* recorded exceeded that of their hosts. In fact, the coevolutionary history of *Anacanthorus* spp. shares some features with those reported in other monogeneans, such as *Lamellodiscus* spp., *Cichlidogyrus* spp., *Dactylogyrus* spp. and *Gyrodactylus* spp. from a range of both marine and freshwater fish [24–28, 56], where, duplications, losses or extinctions were found to represent important evolutionary events. By contrast, in these previous studies, cospeciation was much less common and host switches were more prevalent than in *Anacanthorus* spp. [24, 56].

Host switching is an evolutionary event commonly observed in host-parasite coevolution studies, and has been invoked to justify incongruence between the host and parasite phylogenies [6]. This event has been considered as more costly than cospeciation and duplication, its cost depending on the association studied. For example, in monogeneans that have stages of dispersion chances of host switching would be higher than in parasites that depend on their host for transmission [4]. Host switching can also be more costly due to the putative competition with the species already established and the immune response in the new hosts [57]. So the question why host switching was relatively infrequent in *Anacanthorus* spp. as opposed to most monogeneans studied to date remains open. In the present study, host switching took place only between congeneric fish species within *Salminus* and *Serrasalmus*. These results conform to a scenario of spreading via ecological fitting by resource tracking [57], which allows infections by the same parasite species in different host species without large biological costs for the parasites. Whether these new colonizations would lead eventually to parasite speciation probably depends on the extent of biological and physiological differences between the new and the original hosts [58].

To summarize, the main speciation ways followed by the species of *Anacanthorus* herein studied were duplication and cospeciation. However, cospeciation appeared to be more common than in previous coevolution studies of monogeneans. The present results may just represent a local sample of the speciation pathways within *Anacanthorus* in South America. This genus includes over 70 species and, of the 14 herein studied, eight possibly represent new species to science. Therefore, our knowledge of the diversification of and speciation pathways of *Anacanthorus* is in its infancy. Additional studies of these parasites and their hosts in South America should be promoted, as they might render much needed knowledge about the evolution of one of the most speciose monogenean genera in the Americas.

## References

[1] Desdevises Y. Cophylogeny: insights from fish-parasite systems. Parassitologia. 2007;49:125–28. 18410070

[2] Brooks DR. Testing the context and extent of host-parasite coevolution. Syst Biol. 1979; 28(3):299–307.

[3] Hafner MS, Nadler AS. Phylogenetic trees support the co-evolution of parasites and their hosts. Nature. 1988;332:258–59. doi: 10.1038/332258a0 3347269 10.1038/332258a0

[4] Desdevises Y, Morand S, Jousson O, Legendre P. Coevolution between *Lamellodiscus* (Monogenea: Diplectanidae) and Sparidae (Teleostei): The study of a complex host–parasite system. Evolution. 2002a;56:2459–71. 12583586 10.1111/j.0014-3820.2002.tb00171.x

[5] Santiago-Alarcon D, Rodríguez-Ferraro A, Parker PG, Ricklefs R. Different meal, same flavor: cospeciation and host switching of haemosporidian parasites in some non-passerine birds. Parasit Vectors. 2014;7(286):1–9. 24957563 10.1186/1756-3305-7-286 PMC4077843

[6] Lei BR, Olival KJ. Contrasting patterns in mammal–bacteria coevolution: *Bartonella* and *Leptospira* in bats and rodents”. PLoS Negl Trop Dis. 2014;8(3):e2738 doi: 10.1371/journal.pntd.0002738 24651646 10.1371/journal.pntd.0002738 PMC3961187

[7] Rohde K. A critical evaluation of intrinsic and extrinsic factors responsible for niche restriction in parasites. Am Nat. 1979;114:648–71.

[8] Cone DK, Burt MDB. The host specificity of *Urocleidus adspectus* (Mueller, 1938) (Monogenea:Ancyrocephalinae). J Parasitol. 1982;75:702–6.

[9] Sasal P, Desdevises Y, Morand S. Host-specialization and species diversity in fish parasites: phylogenetic conservatism? Ecography. 1998;21:639–45.

[10] Lambert A, Gharbi SEL. Monogenean host specificity as a biological and taxonomic indicator for fish. Biol Conserv. 1995;72:227–35.

[11] Noble ER, Noble GA, Schad GA, Macinnes AJ. Parasitology. The biology of animal parasites 6th ed. Philadelphia: Lea and Febiger; 1989, 584p.

[12] Poulin R. Determinants of host-specificity in parasites of freshwater fishes. Int J Parasitol 1992;22:753–58. 1428509 10.1016/0020-7519(92)90124-4

[13] Kearn GC. Evolutionary expansion of the Monogenea. Int J Parasitol. 1994;24:1227–71. 7729979 10.1016/0020-7519(94)90193-7

[14] Brooks DR, McLennan DA. Parascript: parasites and the language of evolution Washington, DC: Smithsonian Inst. Press; 1993.

[15] Boeger WA, Kritsky DC. Coevolution of the Monogenoidea (Platyhelminthes) based on a revised hypothesis of parasite phylogeny. Int J Parasitol. 1997; 27(12):1495–511. 9467734 10.1016/s0020-7519(97)00140-9

[16] Rózna L, Tryjanowski P, Vas Z. Under the changing climate: How shifting geographic distributions and sexual selection shape parasite diversification In Morand S; Krasnov B, Littlewood T (eds.). Parasite diversity and diversification: evolutionary ecology meets phylogenetics. Cambridge University Press 2015; pp. 58–76.

[17] Choudhury A, Moore BR, Marques FLP, Kellogg V. Host-switching, and cospeciation: Rescuing straggled ideas. J. Parasitol. 2002;88:5, 1045–48. doi: 10.1645/0022-3395(2002)088[1045:VKHSAC]2.0.CO;2 12435159 10.1645/0022-3395(2002)088[1045:VKHSAC]2.0.CO;2

[18] Banks JC, Paterson AM 2005 Multi-host parasite species in cophylogenetic studies. Int J Parasitol 35(7);741–746. doi: 10.1016/j.ijpara.2005.03.003 15885693 10.1016/j.ijpara.2005.03.003

[19] Conow C, Fielder D, Ovadia Y, Libeskind-Hadas R. Jane: A new tool for the cophylogeny reconstruction problem. Algorithms Mol Biol. 2010; 5:16 doi: 10.1186/1748-7188-5-16 20181081 10.1186/1748-7188-5-16 PMC2830923

[20] Desdevises Y, Morand S, Legendre P. Evolution and determinants of host specificity in the genus *Lamellodiscus* (Monogenea). Biol J Linn Soc Lond. 2002b;77:431–43.

[21] Šimková A, Serbielle C, Pariselle A, Vanhove MPM, Morand S. Speciation in *Thaparocleidus* (Monogenea: Dactylogyridae) parasitizing asian Pangasiid catfishes,” Biomed Res Int. 2013; 1–14. 10.1155/2013/353956 PMC3853038 24350263

[22] Mendlová M, Desdevises Y, Civáňová K, Pariselle A, Šimková A. Monogeneans of West African cichlid fish: evolution and cophylogenetic interactions. PLoS One 2012;7(5): e37268 doi: 10.1371/journal.pone.0037268 22662139 10.1371/journal.pone.0037268 PMC3356412

[23] Mendlová M, Šimková A. Evolution of host specificity in monogeneans parasitizing African cichlid fish. Parasit Vectors. 2014;7(69):1–14. 24529542 10.1186/1756-3305-7-69 PMC3932501

[24] Vanhove MPM, Pariselle A, Van Steenberge M, Raeymaekers JAM, Hablützel PI, Gillardin C, Hellemans B, Breman FC, Koblmüller S, Sturmbauer C, Snoeks J, Volckaert FAM, Huyse T. Hidden biodiversity in an ancient lake: phylogenetic congruence between lake Tanganyika tropheine cichlids and their monogenean flatworm parasites. Sci Rep. 2015;5:13669 doi: 10.1038/srep13669 26335652 10.1038/srep13669 PMC4558575

[25] Šimková A, Morand S, Jobet E, Gelnar M, Verneau O. Molecular phylogeny of con-generic monogenean parasites (*Dactylogyrus*): a case of intrahost speciation. Evolution. 2004;58:1001–18. 15212381 10.1111/j.0014-3820.2004.tb00434.x

[26] Šimková A, Verneau O, Gelnar M, Morand S. Specificity and specialization of congeneric monogeneans parasitizing cyprinid fish. Evolution. 2006;60(5):1023–37. 16817542

[27] Huyse T, Volckaert F. Comparing host and parasite phylogenies: *Gyrodactylus* flatworms jumping from goby to goby. Syst Biol. 2005;54:710–18. doi: 10.1080/10635150500221036 16195215 10.1080/10635150500221036

[28] Hahn C, Weiss SJ, Stojanovski S, Bachmann L. Co-Speciation of the ectoparasite *Gyrodactylus teuchis* (Monogenea, Platyhelminthes) and its salmonid hosts. PLos One. 2015;10(6):e0127340 doi: 10.1371/journal.pone.0127340 26080029 10.1371/journal.pone.0127340 PMC4469311

[29] Thatcher V. E. Amazon fish parasites Sofia: Pensoft; 2006, 508p.

[30] Cohen SC, Justo MCN, Kohn A. South American Monogenoidea parasites of fishes, amphibians and reptiles Rio de Janeiro: Ed. Oficina de Livros; 2013, 663p.

[31] Brandão H, Yamada FH, Toledo GM, Carvalho ED, Silva RJ. Monogeneans (Dactylogyridae) parasitizing gills of *Salminus hilarii* from a Neotropical reservoir, Brazil. Rev Bras Parasitol Vet. 2013;22(4):579–58. doi: 10.1590/S1984-29612013000400020 24473885 10.1590/S1984-29612013000400020

[32] Graça RJ, Ueda BH, Oda FH, Takemoto RM. Monogenea (Platyhelminthes) parasites from the gills of *Hoplias* aff. *malabaricus* (Bloch, 1974) (Pisces: Erythrinidae) in the upper Paraná river floodplain, states of Paraná and Mato Grosso do Sul, Brazil. Check List 2013;9(6): 1484–1487.

[33] Leão MSL, São Clemente SC, Cohen SC. *Anacanthorus toledoensis* n. sp. and *Mymarothecium ianwhittingtoni* n. sp. (Dactylogyridae: Monogenoidea) parasitizing Cage-Reared *Piaractus mesopotamicus* (Characiformes, Characidae) in the state of Paraná, Brazil. Comp Parasitol. 2015;82(2):269–74.

[34] Monteiro CM, Cohen SC, Brasil-Sato MC. New species and reports of dactylogyrids (Monogenoidea) from *Salminus franciscanus* (Actinopterygii: Bryconidae) from the upper São Francisco River, Brazil. Zootaxa. 2015;3941(1):137–43. doi: 10.11646/zootaxa.3941.1.9 25947500 10.11646/zootaxa.3941.1.9

[35] Braga MP, Araújo SBL, Boeger WA. Patterns of interaction between Neotropical freshwater fishes and their gill Monogenoidea (Platyhelminthes). Parasitol Res. 2014;113:481–90. doi: 10.1007/s00436-013-3677-8 24221891 10.1007/s00436-013-3677-8

[36] Van Every LR, Kritsky DC. Neotropical Monogenoidea. *Anacanthorus* Mizelle and Price, 1965 (Dactylogyridae, Anacanthorinae) of piranha (Characoidea, Serrasalmidae) from the Central Amazon, their phylogeny, and aspects of host-parasite coevolution. J Helminthol Soc Wash. 1992;59(1):52–75.

[37] Graça WF, Pavanelli CS. Peixes da planície de inundação do alto rio Paraná e áreas adjacentes Maringá: EDUEM; 2007, 241p.

[38] Takemoto RM, Pavanelli GC, Lizama MAP, Lacerda ACF, Yamada FH, Moreira LHA, Ceschini TL, Bellay S. Diversity of parasites of fish from the upper Paraná river floodplain, Brazil. Braz J Biol. 2009;69(2):691–705. 19738975 10.1590/s1519-69842009000300023

[39] Boeger WA, Husak WS, Martins ML. Neotropical Monogenoidea. 25 *Anacanthorus penilabiatus* n. sp. (Dactylogyridae: Ancyrocephalinae) from *Piaractus mesopotamicus* (Osteichthyes: Serrasalmidae), cultivated in the State of São Paulo, Brazil. Mem. Inst. Oswaldo Cruz. 1995;90:699–70.

[40] Cohen SC, Kohn A, Boeger WA. Neotropical Monogenoidea. 57. Nine new species of Dactylogyridae (Monogenoidea) from the gill of *Salminus brasiliensis* (Characidae, Characiformes) from the Paraná River, State of Paraná, Brazil. Zootaxa. 2012; 3049:57–68.

[41] Bonett MR, Steffen MAA, Trujano-Alvarez L, Martin SD, Bursey CR, McAllister CT. Distribution, abundance, and genetic diversity of *Clinostomum* spp. metacercariae (Trematoda: Digenea) in a modified Ozark stream system. J Parasitol. 2011;97(2):177–84. doi: 10.1645/GE-2572.1 21506775 10.1645/GE-2572.1

[42] Ivanova NV, Zemlak TS, Hanner RH, Hebert PDN. Universal primer cocktails for fish DNA barcoding. Mol Ecol Notes. 2007;7:544–48.

[43] Luton K, Walker D, Blair D. Comparisons of ribosomal internal transcribed spacers from two congeneric species of flukes (Platyhelminthes: Trematoda: Digenea). Mol Biochem Parasitol. 1992;56:323–28. 1484553 10.1016/0166-6851(92)90181-i

[44] Li C, Ortí G. Molecular phylogeny of Clupeiformes (Actinopterygii) inferredfrom nuclear and mitochondrial DNA sequences. Mol Phylogenet Evol. 2007;44: 386–98. doi: 10.1016/j.ympev.2006.10.030 17161957 10.1016/j.ympev.2006.10.030

[45] Rosenthal A, Coutelle O, Craxton M. Large-scale of DNA sequencing templates by microtitre format PCR. Nucleic Acids Res. 1993;21(1):173–74. 8441614 10.1093/nar/21.1.173 PMC309083

[46] Hall T. BioEdit: a user-friendly biological sequence alignment editor and analysis program for Windows 95/98/NT. Nucleic Acids Symp Ser. 1999;41: 95–8.

[47] Edgar RC. MUSCLE: multiple sequence alignment with high accuracy and high throughput. Nucleic Acids Res. 2004;32(5):1792–7. doi: 10.1093/nar/gkh340 15034147 10.1093/nar/gkh340 PMC390337

[48] Tamura K, Stecher G, Peterson D, Filipski A, Kumar S. MEGA6: Molecular evolutionary genetics analysis version 6.0. Mol Biol Evol. 2013;30: 2725–9. doi: 10.1093/molbev/mst197 24132122 10.1093/molbev/mst197 PMC3840312

[49] Darriba D, Taboada GL, Doallo R, Posada D. jModelTest 2: more models, new heuristics and parallel computing. Nat Methods. 2012;9:772. 10.1038/nmeth.2109 PMC4594756 22847109

[50] Stamatakis A. RAxML version 8: a tool for phylogenetic analysis and post-analysis of large phylogenies. Bioinformatics. 2014;30(9):1312–13. doi: 10.1093/bioinformatics/btu033 24451623 10.1093/bioinformatics/btu033 PMC3998144

[51] Lanfear R, Calcott B, Ho SYW, Guindon S. PartitionFinder: Combined selection of partitioning schemes and substitution models for phylogenetic analyses. Mol Biol Evol. 2012;29(6):1695–701. doi: 10.1093/molbev/mss020 22319168 10.1093/molbev/mss020

[52] Legendre P, Desdevises Y, Bazin E. A statistical test for host-parasite coevolution. Syst Biol 2002;51:217–34. doi: 10.1080/10635150252899734 12028729 10.1080/10635150252899734

[53] Balbuena JA, Míguez-Lozano R, Blasco-Costa I. PACo: A novel procrustes application to cophylogenetic analysis. PLoS One. 2013;8(4):e61048 doi: 10.1371/journal.pone.0061048 23580325 10.1371/journal.pone.0061048 PMC3620278

[54] Hutchinson MC, Cagua EF, Balbuena JA, Stouffer DB, Poisot T. PACo: implementing Procrustean Approach to Cophylogeny in R. Methods Ecol Evol. 2017; doi: 10.1111/2041-210X.12736

[55] R Core Team. R: A language and environment for statistical computing R Foundation for Statistical Computing, Vienna, Austria 2015 Available in: http://www.R-project.org/. Accessed in 09/2015.

[56] Míguez-Lozano R, RODRÍGUEZ-GONZÁLEZ A, BALBUENA JA. A quantitative evaluation of host-parasite coevolutionary events in three genera of monopisthocotylean monogeneans. Vie et Milieu. 2017; 67(:2):, in press.

[57] Araújo SBL, Braga MP, Brooks DR, Agosta SJ, Hoberg EP, von Hartenthal FW, Boeger WA. Understanding Host-Switching by Ecological Fitting. PLoS One. 2015;10(10):e0139225 doi: 10.1371/journal.pone.0139225 26431199 10.1371/journal.pone.0139225 PMC4592216

[58] Agosta SJ, Klemens JA. Ecological fitting by phenotypically flexible genotypes: implications for species associations, community assembly and evolution. Ecol Lett. 2008;11:1123–34. doi: 10.1111/j.1461-0248.2008.01237.x 18778274 10.1111/j.1461-0248.2008.01237.x

